# Probabilistic Nested Model Selection in Pharmacokinetic Analysis of DCE-MRI Data in Animal Model of Cerebral Tumor

**DOI:** 10.21203/rs.3.rs-4469232/v1

**Published:** 2024-06-12

**Authors:** Hassan Bagher-Ebadian, Stephen L. Brown, Mohammad M. Ghassemi, Prabhu C. Acharya, Indrin J. Chetty, Benjamin Movsas, James R. Ewing, Kundan Thind

**Affiliations:** 1 Department of Radiation Oncology, Henry Ford Cancer Institute, Detroit, USA; 2 Department of Radiology, Michigan State University, East Lansing, USA; 3 Department of Physics, Oakland University, Rochester, USA; 4 Department of Oncology, School of Medicine, Wayne State University, Detroit, USA; 5 Department of Computer Science and Engineering, Michigan State University, East Lansing, USA; 6 Department of Radiation Oncology, Cedars-Sinai Medical Center, Los Angles, USA; 7 Department of Neurology, Henry Ford Health, Detroit, USA

**Keywords:** Nested Model Selection, Dynamic Contrast Enhanced MRI, Model Averaging, Unsupervised Kohonen Self Organizing Map, Physiological Tissue Characterization

## Abstract

**Purpose:**

Best current practice in the analysis of dynamic contrast enhanced (DCE)-MRI is to employ a voxel-by-voxel model selection from a hierarchy of nested models. This nested model selection (NMS) assumes that the observed time-trace of contrast-agent (CA) concentration within a voxel, corresponds to a singular physiologically nested model. However, admixtures of different models may exist within a voxel’s CA time-trace. This study introduces an unsupervised feature engineering technique (Kohonen-Self-Organizing-Map (K-SOM)) to estimate the voxel-wise probability of each nested model.

**Methods:**

Sixty-six immune-compromised-RNU rats were implanted with human U-251N cancer cells, and DCE-MRI data were acquired from all the rat brains. The time-trace of change in the longitudinalrelaxivity $\left(\Delta R_{1}\right)$ for all animals’ brain voxels was calculated. DCE-MRI pharmacokinetic (PK) analysis was performed using NMS to estimate three model regions: Model-1: normal vasculature without leakage, Model-2: tumor tissues with leakage without back-flux to the vasculature, Model-3: tumor vessels with leakage and back-flux. Approximately two hundred thirty thousand (229,314) normalized $\Delta R_{1}$ profiles of animals’ brain voxels along with their NMS results were used to build a K-SOM (topology-size: 8×8, with competitive-learning algorithm) and probability map of each model. *K*-fold nested-cross-validation (NCV, k=10) was used to evaluate the performance of the K-SOM probabilistic-NMS (PNMS) technique against the NMS technique.

**Results:**

The K-SOM PNMS’s estimation for the leaky tumor regions were strongly similar (Dice-Similarity-Coefficient, DSC=0.774 [CI: 0.731–0.823], and 0.866 [CI: 0.828–0.912] for Models 2 and 3, respectively) to their respective NMS regions. The mean-percent-differences (MPDs, NCV, k=10) for the estimated permeability parameters by the two techniques were: −28%, +18%, and +24%, for $v_{p},K^{\text{trans}}$, and $v_{e}$, respectively. The KSOM-PNMS technique produced microvasculature parameters and NMS regions less impacted by the arterial-input-function dispersion effect.

**Conclusion:**

This study introduces an unsupervised model-averaging technique (K-SOM) to estimate the contribution of different nested-models in PK analysis and provides a faster estimate of permeability parameters.

## 1- Introduction:

In the pharmacokinetic (PK) analysis of brain dynamic contrast-enhanced magnetic resonance imaging (DCE-MRI) data, the principle of parsimony serves as an accepted heuristic^1^. Under the constraint of parsimony, the simplest physiologically meaningful model that sufficiently represents the temporal variation of DCE-MRI data is the best choice for producing stable estimates of model parameters. Parsimonious PK parameters effectively summarize the pathophysiological behavior of the underlying brain tissue by balancing variance and bias in parametric estimates^2,3^. Our research group has introduced a nested model selection (NMS) technique for DCE-MRI analysis in rat and human brains that utilizes an extended Patlak graphical method^2,3^ as the highest-order model. This NMS approach enhances the stability of dynamic contrast enhanced (DCE) MRI data processing^2,3^. However, it relies on the strong assumption that each voxel’s measured time trace of contrast agent (CA) concentration corresponds to a single physiologically nested model. This assumption may produce systematic errors in the parametric estimates; for a given voxel’s time trace of CA concentration, combinations of different NM’s with different contribution levels likely exist.

In this study, we introduce an unsupervised probabilistic nested model selection (PNMS) method based on the model averaging concept for DCE-MRI PK analysis of animal model of cerebral tumor using a Kohonen-Self-Organizing Map (K-SOM) technique. The proposed model is constructed by the concentration-time trace of MR relaxivity change $\left(\Delta {\text{R}}_{1}\right)$ estimated from DCE-MRI information of animal model of brain tumor to perform a PNMS based on the nested model selection results. The PNMS can rank and estimate the uncertainty and probability of different nested models as well as their contribution levels in a given voxel to address the voxel-wise ‘model averaging’ concept that results in a faster estimation of PK parameters.

## 2- Methods:

### 2.1 Imaging and animal population:

Using previously published methods^4^, sixty-six athymic RNU rats were implanted with human U-251N cancer cells to form orthotopic gliomas. A DCE-MRI study (Dual-Echo Gradient-Echo - DGE - 400 acquisitions, with 1.55s apart) tail-vein, with contrast injection (Magnevist 0.25 mmol/kg at undiluted concentration no flush) ~60 sec after the start of the experiment Two T_1_ mapping sequences (TOMROP, T-One-by-Multiple-Read-Out-Pulses) were acquired from all rat brains^5^ before and after the DCE-MRI experiment.

### 2.2 Ethical approval:

All experimental and imaging procedures were conducted at our institution under an protocol approved by the Institutional Animal Care and Use Committee (IACUC) of Henry Ford Health (number 1509) and was performed and reported in compliance with the ARRIVE guidelines^6–8^. Also, all methods were performed in accordance with the relevant guidelines and regulations^6–8^. All animals were anesthetized before and during the MRI experiment using Isoflurane. After all experimental studies were completed, rats were euthanized with an overdose of isofluorane, followed by transcardial perfusion and fixation.

### 2.3 Calculation of Contrast Agent Concentration from DCE-MRI data:

We assume that the gadolinium contrast agent (CA) concentration, [Gd], is proportional to the change in the longitudinal relaxation rate $\left(R_{1}\right)$ after CA administration: $\left[Gd]=C(t)∼\Delta R_{1}(t\right)$, where $R_{1}=1/T_{1}$ and $T_{1}$ is the longitudinal relaxation time. We assume that longitudinal relaxivity for Gd is constant across tissues. Dual Gradient Echo (DGE) imaging allows for the computation of $T_{1}$ and ${T_{2}}^{*}$ as approximately pure and independent components^9,10^. This is essential for the estimation of CA concentration based on $\Delta R_{1}$ and $\Delta {R_{2}}^{*}$. We previously introduced and developed methods for estimating the $\Delta R_{1}$ signal (~ CA concentration) from the DGE pule sequence. As previously noted ^2^, this method introduces equations to describe the measured T_1_-weighted intensities of the first and second echo signals and their relationship to the longitudinal and transverse relaxation times ${\left(T_{1},T_{2}\right)}^{*}$, repetition time $\left(T_{R}\right)$, echo time ($\left.T_{E}\right)$, flip angle (θ), and equilibrium longitudinal magnetization $\left(M_{0}\right).{T_{2}}^{*}$ includes the effects of both static dephasing and irreversible dephasing. In this study, the voxel-wise profiles of the CA concentration map, $\Delta R_{1}(t)$, were directly estimated from the DCE-MRI experiment, and the pre and post $T_{1}$ maps estimated by the preand post-DCE $T_{1}$ pulse sequences (T One by Multiple Read Out Pulses, TOMROP/Look-locker^11^). The time trace of change in the longitudinal relaxation time, $\Delta R_{1}$ in all the voxels of the animal’s brain, corresponding and proportional to the time trace of contrast agent concentration, for 66 rat brains’ DCE-MRI studies were calculated^3^.

### 2.4 Post-processing and Conventional Nested Model Selection in PK Analysis:

Post-processing and pharmacokinetic compartmental analyses of DCE-MRI data were carried out following published methods^2,3^. We used a nested model selection (NMS)^2,3,12^ paradigm based on Patlak and extended-Patlak graphical methods^13–15^. As illustrated in subfigure 1A, we have shown^2,3^ that three physiologically nested models can be derived from the standard model to describe possible physiological conditions of underlying tissue pathology^2,3^. We have generated a series of stable processing pipelines accordingly, to produce vascular parametric maps based on the NMS technique^2,3^. As shown in subfigure 1A, the NMS method^2,3^ was used to generate maps of brain regions labeled with the number of permeability parameters used to describe the data^2^: ***a)*** Model 1 region: normal vasculature with no leakage, the only parameter estimated is plasma volume, $v_{p}$; ***b)*** Model 2 region: tumor tissues with CA leakage without measurable back-flux to the vasculature, in which case $v_{p}$ and, $K^{\text{trans}}$ can be estimated; or ***c)*** Model 3 region: tumor vessels with CA leakage and measurable back-flux and, thus, $v_{p},K^{\text{trans}}$, and $k_{ep}$, or extracellular extra-vascular volume, $v_{e}$ (ratio of $K^{\text{trans}}$ and $K_{ep}$) can be estimated. Three model equations representing the three physiologically nested models were constructed as follows (Equations 1–3) and used for the conventional PK NMS analysis:

(1) $$C_{\text{tissue}}(t)=\left[v_{p}C_{AIF}(t)\right]$$

(2) $$\begin{array}{rr} & C_{\text{tissue}}(t)=\left[v_{p}C_{\text{AIF}}(t)+K^{\text{trans}}\int_{0}^{t}C_{AIF}(\lambda )d\lambda \right] \end{array}$$

(3) $$C_{\text{tissue}}(t)=\left[v_{p}C_{AIF}(t)+K^{\text{trans}}\int_{0}^{t}C_{AIF}(\lambda )e^{-K_{ep}(t-\lambda )}d\lambda \right]$$

Where $C_{\text{AIF}}(t)$ *and* $C_{\text{tissue}}(t)$ refer to the contrast agent (CA) concentration measured from plasma (or arterial input function, AIF) and tissue of interest in the brain, respectively. Hematocrit ratio $\left(H_{ct}\right)$ was not included in the equations. The assumption is that the CA is excluded from the erythrocytes of blood, and that the change in $R_{1}$ is proportional to the concentration of CA in plasma. Since we are estimating plasma volume, there’s no correction warranted for $H_{ct}$.

To allow the spin system to reach a true equilibrium after the start of the highly saturated DCE-MR imaging, the first 20 timepoints of the pre-injection part of the signals were excluded from the analysis (about 30 s).

### 2.5 Model Development and Validation:

A total of 229,314 normalized profiles (149668, 60268, and 19378 profiles for Models 1, 2 and 3, respectively) were extracted from the rat brains’ voxels and used to construct and validate the unsupervised K-SOM method^16,17^.

An unsupervised machine learning technique, A K-SOM^16,17^ or self-organizing feature map, was used to produce a low-dimensional representation of higher dimensional data set with complex structures while preserving the topological structure of the data. During the K-SOM analysis, a competitive learning algorithm^18–20^ along with Best Matching Unit (BMU) strategy were employed to identify the “winner” nodes/neurons for an 8×8 topology. The cover steps for initial covering of the input space for ordering the phase steps of the K-SOM was set to 100. The K-SOM’s architecture was hexagonal with an initial neighborhood size of three, and the maximum epoch was set to 250 epochs for batch training mode. A BMU-based hit map was generated for the CA concentration profiles within the animal brains. The model choice labels for all three models (at the Confidence Level of CL=95%) estimated from the conventional PK-NMS analyses for all profiles were used as the source of truth for the conventional nested models (with high Confidence Level: 95%) to calculate the tagged BMUs on the K-SOM topology space. The hit maps and their corresponding labels were used to estimate the three K-SOM probability and iso-probability maps for the three different model choices.

Dice Similarity Coefficients (DSCs)^21^ for different model regions generated by the conventional NMS analysis and the K-SOM probabilistic NMS (at NMS probabilities of 50%) were used to evaluate the performance of the trained K-SOM and the conventional NMS on DCE-MRI data. The calculated DSCs compared the similarity of the model regions estimated by the PNMS technique against the physiological state identified by the conventional NMS method.

To investigate and validate the performance of the constructed K-SOM to perform the probabilistic NMS on DCE-MRI data for the three physiologically nested models, a k-fold (k=10) Nested Cross Validation (NCV) analysis^22^ was performed. The full dataset for all the three models was randomly permuted (using Random Permutation Sampling, RPS^23^) and split into 10 non-overlapping folds^24^ (ratio for training/test: 0.66/0.34, 44/22^24^). Two independent loops were defined as outer and inner loops. In the outer loop, for each epoch, the data was split into two folds (training + validation fold, and a test fold), and for the inner loop, only the validation + training fold was used to construct a series of K-SOMs. Then, for each iteration, an unsupervised K-SOM was constructed in the inner loop using the training cohorts. The trained K-SOM constructed in each fold of the inner loop was used as the PNMS predictor of the test cohorts of its respective fold in the outer loop. This process was repeated 10 times (k=10) and at each repetition, an independent test set was withheld for the estimation of the performance of the constructed K-SOM PNMS model of the inner loop for different nested models and their respective permeability parameters.

In each iteration, the model 1 region estimated by the PNMS at 50% threshold was used to mask-out the whole brain to identify the two model regions (Models 2 and 3) of the rat brains. Then, two DSCs were calculated and averaged (over the 10 folds) for the model 2 and 3 regions estimated by the conventional NMS (at the Confidence Level of 95%) and K-SOM PNMS techniques. Finally, for different model regions, three permeability parameters were estimated and averaged over the 10-fold nested cross validation for the two techniques and their mean percent differences (MPD) were calculated. The voxel-wise permeability parameters, $v_{p}(x,y,z),K^{\text{trans}}(x,y,z)$, and $v_{e}(x,y,z)$, for the K-SOM PNMS technique were calculated as follows:

(4) $$v_{p}\left(x,y,z\right)=\frac{P_{1}\left(x,y,z\right).v_{p}^{\left(M1\right)}\left(x,y,z\right)+P_{2}\left(x,y,z\right).v_{p}^{\left(M2\right)}\left(x,y,z\right)+P_{3}\left(x,y,z\right).v_{p}^{\left(M3\right)}\left(x,y,z\right)}{P_{1}\left(x,y,z\right)+P_{2}\left(x,y,z\right)+P_{3}\left(x,y,z\right)}$$

(5) $$K^{\text{trans}}(x,y,z)=\frac{P_{2}\left(x,y,z\right).K^{\text{trans}\left(M2\right)}\left(x,y,z\right)+P_{3}\left(x,y,z\right).K^{\text{trans}(M3)}(x,y,z)}{P_{2}(x,y,z)+P_{3}(x,y,z)}$$

(6) $$v_{e}(x,y,z)=\frac{K^{\text{trans}}(x,y,z)}{K_{ep}^{\left(M3\right)}(x,y,z)}$$

where $P_{1}(x,y,z),P_{2}(x,y,z)$, and $P_{3}(x,y,z)$ refers to the voxel-wise probabilities for the three nested models: models 1, 2, and 3, respectively. The $v_{p}^{M1}(x,y,z)$, $v_{p}^{M2}(x,y,z)$, and $v_{p}^{M3}(x,y,z)$ in Equation 1 refers to the estimated voxel-wise plasma volumes for models 1, 2, and 3, using the conventional NMS technique. The $K^{\text{trans}(M2)}(x,y,z)$, and $K^{\text{trans}(M3)}(x,y,z)$ in Equation 2 refers to the voxel-wise forward transfer constants estimated for the nested models 1 and 2 using the conventional NMS technique. The $K_{\text{ep}}^{M3}(x,y,z)$ in Equation 3 refers to the voxel-wise reverse transfer constant for model 3 using the conventional NMS technique.

## 3- Results:

Subfigure 1A illustrates three physiologically nested models, their vascular and extra-cellular extravascular compartments, and their estimable vascular parameters according to the concept of conventional NMS technique. Subfigure 1B shows graphs of typical time-traces of the CA concentration $\left(\Delta R_{1}\right)$ computed for the three nested models according to the observation equations (Equations 1–3) for typical permeability parameters. Subfigure 1C demonstrates the concept of the model averaging technique for estimation of the probabilistic nested model selection from the conventional NMS results for a typical voxel. Subfigure 2A and 2B illustrate the first and second echo of the DCE-DGE for a slice of rat brain about 8 minutes after the tail vein administration of CA. Subfigure 2C–F demonstrates the model choice map and its corresponding permeability parameter maps ($v_{p},K^{\text{trans}}$, and $v_{e}$) calculated by the conventional NMS technique for the same slice of rat brain. Subfigure 3A illustrates the normalized K-SOM hit map (BMU) constructed from 66 rat brains’ voxels. Subfigure 3B demonstrates the topology space of the trained K-SOM network for the three nested model probabilities on the feature space. Subfigures 3C to 3F show the magnified versions (zoomed by factor of 4 for a better visualization) of the K-SOM feature space probabilities for the nested models 1, 2, 3, and their fused map (coded in RGB colors) respectively. Subfigure 4A, 4C, and 4E illustrates the trained K-SOM probability feature spaces for different nested models with their masked-out regions (estimated at the 50% probability threshold for each model, identified by dark neurons) for the three nested models. Subfigure 4B, 4D, and 4F demonstrate the normalized ΔR1 averaged over the typical rat brain’s regions corresponding to the selected neurons/nodes (dark neurons) on the K-SOM probability space for Models 1, 2, and 3, respectively. The K-SOM based PNMS and conventional NMS analyses results for six slices of different rat brains are shown in Figure 5. The six columns (from left to right) of Figure 5 demonstrate the K-SOM normalized hit map (BMU), K-SOM probability maps for the three models (Model 1, 2, and 3), K-SOM fused probability maps (coded in RGB colors) for the three nested models, and the conventional NMS map (at 95% Confidence Level), respectively. Table 1 shows the model-based permeability parameters estimated by the conventional NMS technique and the K-SOM PNMS method for the NCV (k=10). The last column of the table shows the mean percent difference of each parameter between the two techniques: 100x(PNMS-NMS)/NMS. The average Dice Similarity Coefficients (NCV, k=10 folds) for the leaky tissues were 0.774 [CI: 0.731–0.823], and 0.866 [CI: 0.828–0.912] for Models 2 and 3, respectively. The average plasma volumes (NCV, k=10) estimated by the conventional NMS and PNMS techniques were: 2.134% [CI: 1.942%, 2.369%], and 1.573% [CI: 1.429%, 1.629%], respectively. The MPD for the plasma volume was −28.004%. The average forward volumetric transfer constant (NCV, k=10) estimated by the conventional NMS and PNMS techniques were: 0.140 min^−1^ [CI: 0.131 min^−1^, 0.169 min^−1^], and 0.165 min^−1^ [CI: 0.153 min^−1^, 0.175 min^−1^], respectively. The MPD for the forward volumetric transfer constant was +18.157%. The average interstitial space (NCV, k=10) estimated by the conventional NMS and PNMS techniques were: 13.649% [CI: 12.282%, 15.287%], and 16.949% [CI: 15.424%, 18.136%], respectively. The MPD for the interstitial space was +24.179%.

## 4- Discussion:

The K-SOM analysis technique used in this study preserved and mapped the dynamic characteristics of model-based $\Delta R_{1}$ information at different time points to a feature space, and also correspondingly compared the information of different time points during the mapping process using a competitive learning approach^18–20^. The K-SOM technique is an unsupervised learning method which enables detection and preservation of the topological relationship of the training dataset based on the information similarity. The main goal of the study was to evaluate and compare the voxel-wise pathophysiological information content of the DCE-MRI information, such as distribution of the different NM information, their similarities, uncertainty levels, and model diffusivity on the feature space to perform probabilistic model selection of PK data from rat brain tumors.

We investigated the feasibility of using a K-SOM technique to perform probabilistic *model averaging* on the DCE-MRI information profiles that are directly associated with tissue response $\left(\Delta R_{1}\right)$ compared to its conventional model selection technique to calculate the probability of different pharmacokinetic-based pathophysiological states of tissues according to the nested model selection theory.

In this study, recruitment of such an unsupervised mapping revealed different degrees of similarities and associations^16,17^ among model-based dynamic information extracted from tumor and surrounding normal tissues with their respective pathophysiological states. The K-SOM probabilistic NMS concept proposed in this study allows for a selection of an optimal PK nested model, weighted with a similarity-based model information using the *‘model averaging’* concept that produces a less biased estimate of permeability parameters best fitted to the DCE-MRI information^2,3^. Once constructed, the K-SOM-based PNMS (works based on the model averaging concept) in this study results in faster characterization of tumor physiology, microvasculature and microenvironmental parameters.

As shown in subfigure 3B, there are three dominant clusters on the K-SOM feature space (dark blue, dark green, and dark red) associated with the three conventional nested models. The K-SOM’s neurons on the feature space with lighter colors and mixture of the shades of the three main colors (blue, green, and red) in adjacent to these dominant clusters are responsible for characterization of the voxels’ temporal information that may contain more than one single physiologically nested model with a higher degree of vasculature heterogeneity. These voxels may contain a combination of normal and leaky vasculatures.

We assumed that the change in the longitudinal relaxation rate $\left(R_{1}\right)$ after CA administration is proportional to the contrast agent (CA) concentration,: $\left[Gd]=C(t)\sim \Delta R_{1}(t\right)$, where $R_{1}=1/T_{1}$ and $T_{1}$ is the longitudinal relaxation time. Indeed, this assumption is well supported in most DCE-MRI studies because they are conducted under the conditions of rapid repetition time and Ernst tip-angle adjusted for a mean decrease in $R_{1}$. Under these conditions, studies in multicompartmental systems demonstrate little effect of water exchange mechanisms^25,26^.

**Figure 4:**
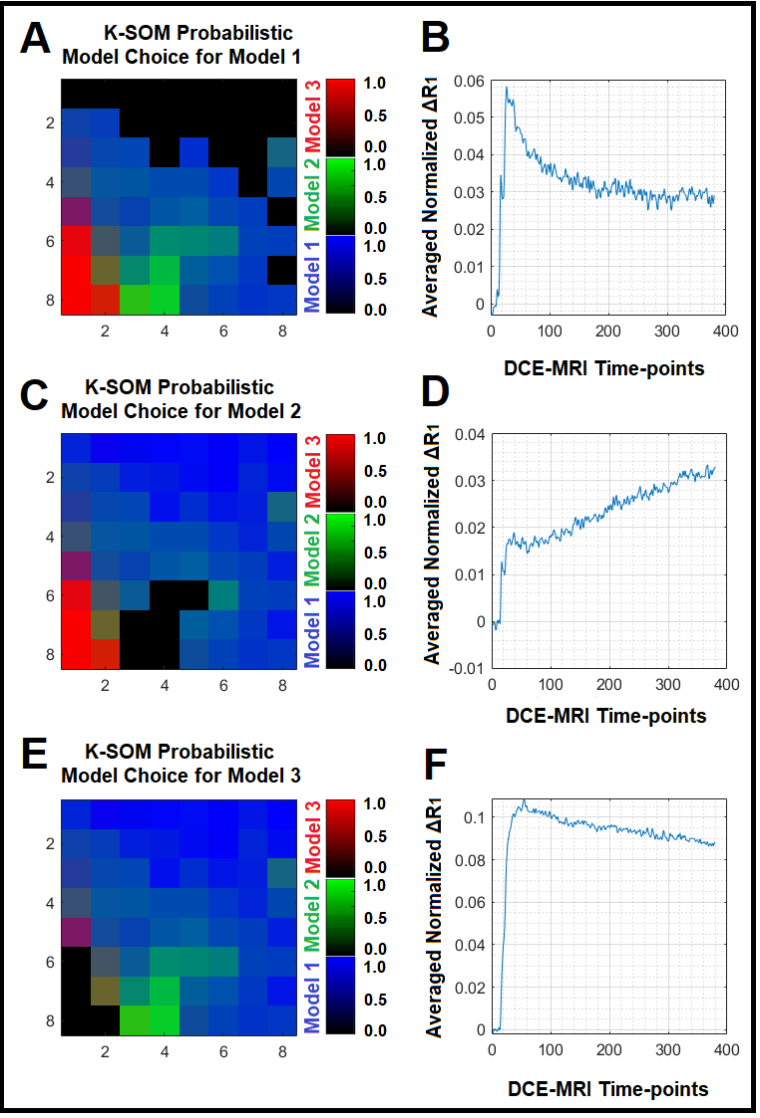
Subfigure 4A, 4C, and 4E illustrate the trained K-SOM probability feature spaces for the three nested models with their masked-out regions (estimated at the 50% probability threshold for each model, dark neurons) for the three nested models. Subfigure 4B, 4D, and 4F demonstrate the normalized $\Delta R_{1}$ averaged over the typical rat brain’s regions corresponding to the selected neurons/nodes (dark neurons) on the K-SOM probability space for Models 1,2, and 3, respectively.

We examined the information content of the relaxivity change $\left(\Delta R_{1}\right)$ measured from a rat brain’s voxel using an unsupervised K-SOM algorithm^16,17,27,28^ to estimate the probability of the existence of different nested models within that voxel. The time traces of relaxivity change $\left(\Delta R_{1}\right)$ were channelized into three dominant models with high certainties (CL=95%) using the conventional NMS technique and then projected on a two-dimensional topology space to reveal any potential non-linearities, similarities, and dissimilarities of the spatiotemporal structures of the $\Delta R_{1}$ profiles in the form of clusters on the K-SOM’s feature space. The notable characteristic of K-SOM method is that the detailed structures of input vectors $\left(\Delta R_{1}\right)$ that are close, and similar in high dimensional space are mapped to nearby nodes in its topology space. It is in essence, an unsupervised method for data dimensionality reduction and grouping the information, as it maps high-dimension inputs to a low dimensional discretized representation while conserving the underlying structures of its input space^16,17,27,28^. This generated distinct clusters with different levels of scattered fragments of $\Delta R_{1}$ information without any supervision on the K-SOM’s feature space. The blue, green, and red zones on the fused K-SOM’s topology maps (see subfigure 3B) correspond to the models 1, 2, and 3 (estimated from the conventional NMS PK analysis with CL=95%), respectively. The three dominant clusters in this figure strongly confirms the value and discriminant power of the $\Delta R_{1}$ information for revelation and capture of the nested models’ features for characterization of tumor heterogeneity and microvascular characteristics according to the conventional NMS concept. As shown in Figure 4, the three dominant clusters associated with the three conventional nested model regions on the K-SOM’s feature space produced three average $\Delta R_{1}$ profiles (subfigures 4B, 4D, and 4F) that are strongly in agreement with their respective graph of $\Delta R_{1}$ profiles (see subfigure 1B) generated by the Equations 1–3.

**Figure 5:**
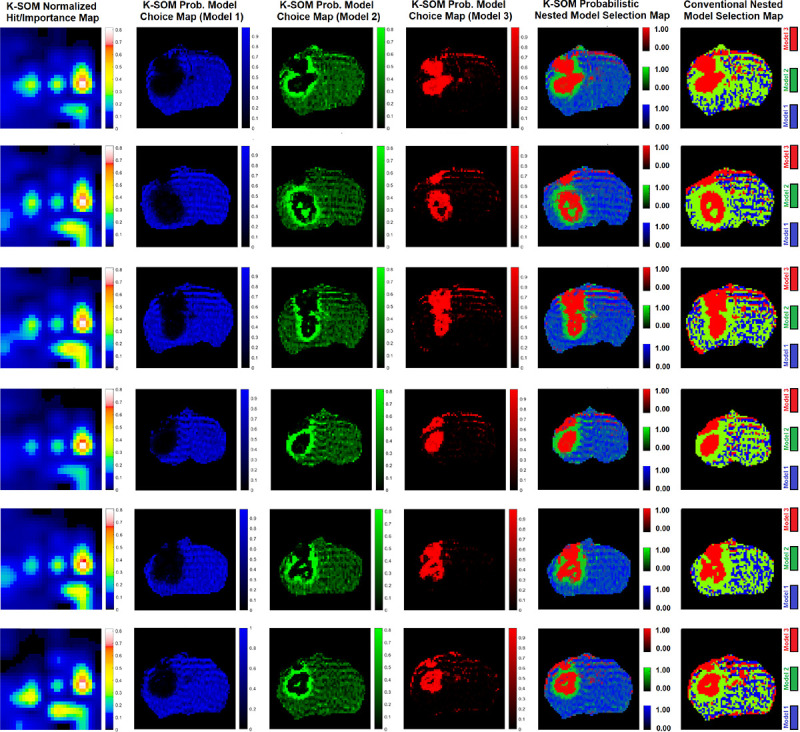
The K-SOM based PNMS and conventional NMS analysis results for six slices of different rat brains are shown in this figure. The six columns (from left to right) of this figure demonstrate the K-SOM normalized hit map, K-SOM probability map for Model 1, K-SOM probability map for Model 2, K-SOM probability map for Model 3, K-SOM fused probability maps for all three models, and the conventional NMS map (at 95% Confidence Level), respectively.

As shown in Figure 5, the K-SOM PNMS has produced stable maps of nested model regions with smooth transitions on their borders. The Model-1 regions (corresponding to non-leaky normal tissues) estimated by the K-SOM PNMS technique are less impacted by the dispersion effects due to the arterial input function and magnetic field gradient. This leads to a lower mis-classification for Model-1 and 2 regions. This is because the K-SOM efficiently captures the detailed characteristics of the $\Delta R_{1}$ profiles and their contribution levels within the normal tissues’ voxels.

The results of this study confirm that the K-SOM PNMS technique can efficiently generate probabilistic nested model maps with less-biased estimates of their respective permeability parameters compared to the conventional NMS technique. Since the K-SOM was trained with the tissue response $\left(\Delta R_{1}\right)$ information, this will preserve the properties or the potential pathophysiological structures of the DCE MRI data that are similar across rat and human brains for more robust modeling. Since the physiological properties of brain tissue are mostly constant across species and pathologies^29–31^, the associations of tissue response information with brain tissue physiology (NMS information) revealed in this study can be expected to reliably scale and translate to human Glioblastoma (GBM) to estimate the physiological properties of solid tumors (such as glioblastoma, GBM) and soft surrounding normal tissues in embedded tumors of humans.

The average DSC values for the two models (DSC= 0.774 [CI: 0.731–0.823], and 0.866 [CI: 0.828–0.912] for Models 2 and 3, respectively) along with permeability parameters evaluated and measured from the k-fold NCV (k=10) technique strongly confirms the convergence between the two methods (conventional and probabilistic NMS techniques) at higher level of certainties (NMS at CL=95% and PNMS at 50% or higher threshold).

The k-fold NCV analysis performed in this study, reveals the information content of different model-based $\left(\Delta R_{1}\right)$ profiles and their stabilities to describe the three pathophysiological states of the tissue. As shown in Table 1, the average values of the permeability parameters reported for the outer loops of the k-fold NCV (k=10) are less (the MPDs for the three permeability parameters were: −28%, +18%, and +24% for $v_{p},K^{\text{trans}}$, and $v_{e}$, respectively) than the values estimated by the conventional NMS technique. Interestingly, the variations (all confident intervals) of the permeability parameters estimated by the KSOM-PNMS are less than their values estimated by the conventional NMS technique.

The $\Delta R_{1}$ profiles estimated in this study can be susceptible to noise, arterial input function dispersion or delay^32,33^, contrast agent arrival time differences among different animals, etc. These systematic and random effects can result in uncertainties in the classification models, both conventional and probabilistic NMS techniques. For the other *known* sources of systematic error, a data-driven approach to model selection will minimize over- or under-fitting to these parameters^2,3^. Of note, if the data has systematic errors due to measurement, data-driven approaches will likely be limited.

It is clear from an examination of the data that there are elements in modeling DCE-MRI that are not accounted for (*c.f. figure 8 of Ewing and Bagher-Ebadian2*) and cannot be accounted for if over-fitting is to be avoided. These *‘tapering effects’* are typical in modeling of all biological systems^1^. As long as effects due to T2* dephasing are accounted-for in the acquisition, the two most evident sources of error are errors in estimating the true arterial contrast agent concentration, and dispersion due to flow of the arterial input function (AIF) as it progresses from the large arteries to the capillary bed, where exchange with the extravascular space takes place. Estimating the true AIF concentration presents significant difficulties, with inflow, outflow, and partial-volume effects undermining efforts at the site of measurement.

This, and dispersion in the arterial tree^34,35^ substantially undermine the assumption that either the amplitude or the shape of the tissue input function can be determined from a time trace of arterial contrast. One approach to estimating input amplitude is to employ a group-averaged time trace of arterial CA concentration normalized to a known vascular volume in normal brain tissue (putamen)^2,36^, thus deliberately introducing a bias that is presumably smaller than the bias of measuring contrast change directly from a large artery and inferring dynamic contrast agent concentration. This does not address problems with AIF dispersion. Therefore, quantification of all these tapering effects and estimation of their impacts on the raw DCE-MRI signal and their propagations into the tissue response is very complex and hard to simulate for the validation of the NMS and K-SOM PNMS techniques. As future works, investigation of these systematic and random effects on the performances of the conventional and probabilistic NMS techniques is warranted.

One of the first self-organizing algorithms^27^, the K-SOM algorithm groups multi-dimensional data in the form of clusters on feature space with no supervision of the intent of visualization, clustering analysis, and dimensionality reduction^16–20,27,37^. Nonlinear dimensionality reduction, unsupervised learning, intuitive clustering relationship, feature space visualization, easy interpretation, flexibility to work with numerical, categorical, and discrete datasets as well as robustness to the noise of the signal are the most important benefits of using the K-SOMs^38^. Many improvements^27,37^ have been proposed to the original architecture, which include the Batch-SOM^27^, Dot-Product-SOM^27^, a SOM that focuses on identifying a linear combination of model vectors instead of winner nodes^28^. For instance, the O(log_2_M)-SOM^39^ utilizes a stratification technique that inherently deals with propagation to neighborhood nodes on the feature space. The main goal of these evolved versions of the K-SOM is to prioritize and improve the optimization and representation of feature information within the feature space. Other algorithms that can also improve the original K-SOM’s performance, stability, convergence, and its adaptability with other problems include Growing Grid SOM^40^, Growing Neural Gas^41^ algorithms, and Hierarchical-SOM^42^. These algorithms consist of an additional layer of nodes to automatically determine the optimal topology size of the K-SOM based on the properties and cost functions determined by the original algorithm. Despite the promising advantages provided by these modified K-SOM algorithms over its original version such as memory and speed optimizations, as well as improved data representation, they may be susceptible to bias in generating clusters and in the quality of the feature visualization on the feature space. In this study, the K-SOM’s topology size was selected as 8X8 that can produce a maximum of 64 distinctive clusters on the image space. Choosing a larger size of the K-SOM’s topology would increase the precision of the estimated probabilities for different models. However, as the size of the network increases, the chance of the network running into an over-fitting condition and capturing more detailed information that could be irrelevant to the meaningful spatiotemporal trend of the pathophysiological state of the tissue increases. Thus, further investigation of the effects of different K-SOM’s architecture, topology sizes, the recruited algorithms, its comparison with different unsupervised models such as AutoEncoders^43,44^, as well as other model averaging techniques such as Akaike’s Information Criteria (AIC)^12,45^, AIC corrected (AICc), Bayesian information criteria (BIC)^46,47^ and their impact on the model efficiency for probabilistic NMS analysis of DCE-MRI data is warranted^12,48^.

This study demonstrates an application of an adaptive unsupervised model to improve robustness and computation speed of the NMS technique that appear in conventional approaches to estimate DCE-MRI vascular parameters. Compared to conventional NMS methods, the trained K-SOM PNMS technique is computationally faster and takes a fraction of a second (Core (TM)-6700HQ, CPU @2.60 Hz) to calculate all the probabilistic NMS maps for an entire 3D DCE-MRI brain volume (for entire animal brain with 3 full slices).

## 5- Conclusions:

This work establishes an important first step toward spatiotemporal MR characterization of brain and brain tumor regions using a probabilistic nested model selection technique. This is fundamental toward an accurate estimation of vasculature parameters critical in staging tumors, evaluating early tumor response to treatments, as well as designing and optimizing DCE-MR imaging for precise characterization of brain tumors.

## Figures and Tables

**Figure 1:**
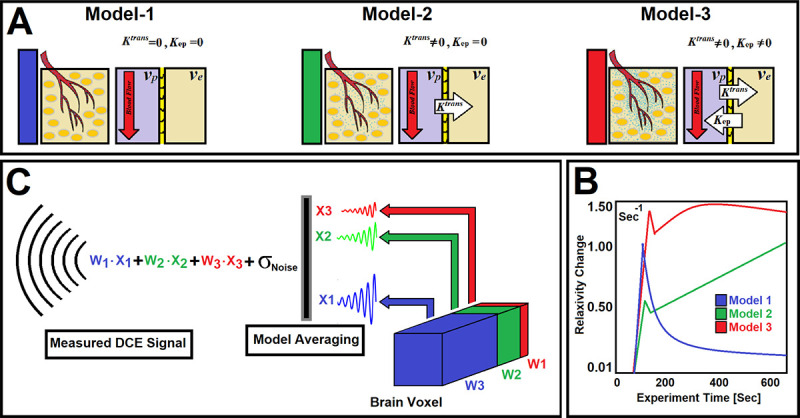
Subfigure 1A illustrates three physiologically nested models, their vascular and extra-cellular-extra vascular compartments, and their estimable vascular parameters. Subfigure 1B shows typical time-traces of the CA concentration computed for the three nested models according to the observation equations (Equations 1–3) for typical permeability parameters. Subfigure 1C demonstrates the concept of the model averaging technique for estimation of the probabilistic nested model selection from the conventional NMS results for a typical voxel.

**Figure 2:**
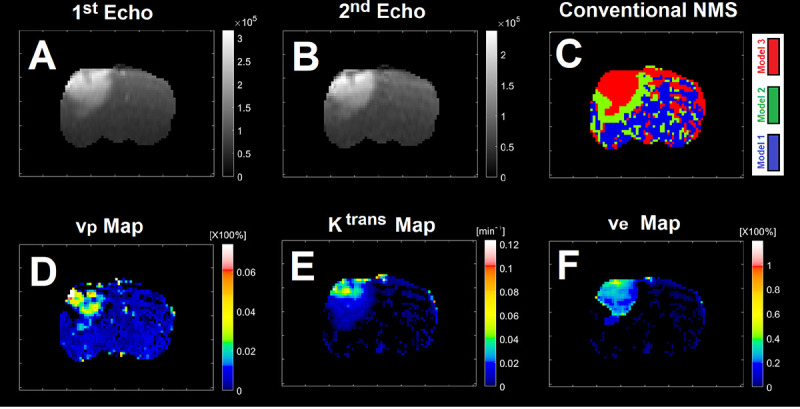
Subfigure 2A and 2B illustrate the first and second echo of the DCE-DGE for a slice of rat brain about 8 minutes after the tail vein administration of CA. Subfigure 2C–F demonstrate the model choice map and its corresponding permeability parameter maps ($v_{p},K^{\text{trans}}$, and $v_{e}$ maps) for the same slice of rat brain.

**Figure 3:**
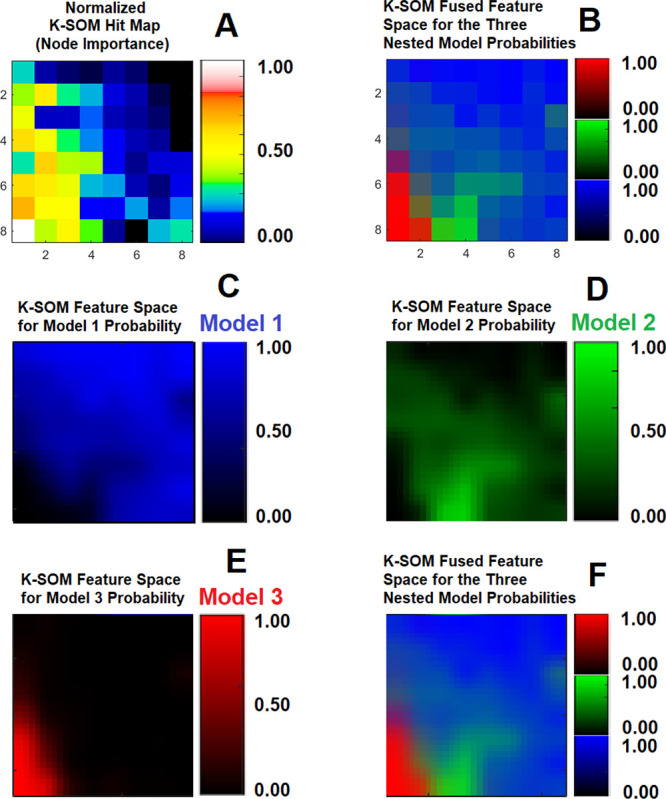
Subfigure 3A illustrates the normalized K-SOM hit map constructed from 66 rat brain’s voxels. Subfigure 3B demonstrates the topology of the trained K-SOM feature space for the three nested model probabilities. Subfigures 3C to 3F show the magnified versions (for better visualization) of the K-SOM feature space probabilities for Models 1, 2, 3, and their fused map respectively.

**Table-1:** This table shows the average permeability parameters estimated by the conventional NMS technique and the K-SOM probabilistic nested model selection method using the k-fold NCV (k=10). The last column of the table shows the mean percent differences (100×[PNMS estimate-NMS estimate)/NMS estimate]) of the PNMS’s estimates compared to their baseline values (NMS).

	Conventional NMS			Probabilistic NMS			
	Mean	(CI-LB)	(CI-UB)	Mean	(CI-LB)	(CI-UB)	MPD [%]
**Plasma Volume [%]**	2.134	1.942	2.369	1.537	1.429	1.629	−28.004
**Forward Volumetric Transfer Constant [1/min]**	0.140	0.131	0.169	0.165	0.153	0.175	18.157
**Extra Cellular Extra Vascular Volume [%]**	13.649	12.284	15.287	16.949	15.424	18.136	24.179

## Data Availability

All imaging data used in this investigation along with programming codes and results are available and can be shared upon request to Drs. Bagher-Ebadian, Ewing, and Brown.
